# Drop‐out rates in animal‐assisted psychotherapy – Results of a quantitative meta‐analysis

**DOI:** 10.1111/bjc.12492

**Published:** 2024-08-05

**Authors:** Linnea Seeger, Andrea Kübler, Kirsten Hilger

**Affiliations:** ^1^ Department of Psychology I Würzburg University Würzburg Germany

**Keywords:** animal‐assisted psychotherapy, commitment, drop‐out, mental disorders, meta‐analysis

## Abstract

**Introduction:**

Animal‐assisted psychotherapy is an emerging field with great potential and growing popularity. However, empirical research on its effectiveness is insufficient, and consistent evidence about patients' commitment is missing. The present meta‐analysis addresses this gap by systematically comparing drop‐out rates in animal‐assisted psychotherapy and by relating the resulting across study drop‐out rate to across study drop‐out rates reported in meta‐analyses on conventional psychotherapy.

**Method:**

Fifty‐seven studies published until August 2022 were identified as eligible for meta‐analytic comparison, that is, they conducted animal‐assisted psychotherapy on at least one group of psychiatric patients and reported drop‐out rates. Potential moderating influences of the type of animal and patients' disorder were considered, as well as multiple other demographic and study design variables.

**Results:**

The across study drop‐out rate in animal‐assisted psychotherapy was 11.2%. This was significantly lower than the across meta‐analyses drop‐out rate of conventional psychotherapy (*d* = −.45, *p* = .0005). Although effects of moderator variables could not be evaluated statistically due to too small and heterogeneous data sets, descriptive results suggest influences of the type of animal and patient disorder. However, study quality ratings identified serious shortcomings regarding proper research design, most critically concerning the report of effect size measures, the use of standardized intervention plans and Open Science practices.

**Conclusion:**

Drop‐out constitutes a major problem of psychotherapeutic research and practice. By proposing that the inclusion of an animal in the psychotherapeutic setting can enhance patients' commitment and by outlining challenges and opportunity of animal‐assisted psychotherapy, this meta‐analysis offers a starting point for future research in this evolving field.

**Practitioner points**

- We systematically evaluate empirical evidence on animal‐assisted psychotherapy to shed light on the question whether the inclusion of animals in psychotherapeutic settings may serve as a means to increase patients' commitment, lower drop‐out rates, and therefore enhance the effectiveness of therapy.
- Our results propose animal‐assisted therapy as valuable add‐on to standard psychotherapy that can be beneficial in clearly defined cases. In those cases, therapeutic animals may render therapy effective that may otherwise fail.
- We discuss practical challenges of animal‐assisted psychotherapy and evaluate factors that may determine whether therapy will benefit of including an animal or not.

## INTRODUCTION

### Definition and effectiveness of animal‐assisted psychotherapy

Animal‐assisted (psycho‐)therapy describes ‘the therapeutic use of pets to enhance individuals’ physical, social, emotional or cognitive functioning' (American Psychological Association, 2023) and has gained increasing importance in recent years (Bachi, 2012; Jones et al., 2019; Lentini & Knox, 2015; Schramm et al., 2015). Although the application of animal‐assisted interventions in psychological treatment has a long history of more than 60 years (Lentini & Knox, 2009; Morrison, 2007; Nimer & Lundahl, 2007; Seitz et al., 2017; Serpell et al., 2017) and research on effects of animals on human health in other contexts such as physical therapy and rehabilitation is convincing (Kendall et al., 2015), research on the effectiveness of animal‐assisted psychotherapy is still in the early stages (Burton et al., 2019). Inconsistent results (Cirulli et al., 2011; Germain et al., 2018) and insufficient study quality are frequently preventing sound conclusions (Kamioka et al., 2014; Lee et al., 2016; O'Haire, 2013; Schramm et al., 2015).

Nevertheless, there seems to exist at least some consensus that animal‐assisted interventions can be effective for the treatment of patients with mental disorders (Kendall et al., 2015) with even long‐lasting effects (Nurenberg et al., 2015). This is especially true for patient populations that have not responded to other therapeutic approaches (Ewing et al., 2007) as well as for children and adolescents (Lee et al., 2016). Finally, large‐scale meta‐analyses concluded that animal‐assisted therapy is a helpful adjunct to conventional therapy, magnifying its effects on symptoms and social interaction (Nimer & Lundahl, 2007; Selby & Smith‐Osborne, 2013).

### Mechanisms of animal‐assisted interventions

Jones et al. (2019) describe two mechanistic pathways of animal‐assisted psychotherapy. On the one hand, contact with animals directly reduces symptoms (e.g., stress, anxiety), and on the other hand, therapy‐relevant variables, such as motivation and acceptance are positively influenced through the improvement of the therapeutic process. In this regard, better trust‐building, improved therapeutic relationships (Balluerka et al., 2015; Ewing et al., 2007; Karol, 2007; McCullough et al., 2015; Redefer & Goodman, 1989), and a generally more positive attitude towards the therapist (Schneider & Harley, 2006) have been observed when animals are present. Improved therapeutic alliance (Wesley et al., 2009) and increased motivation (Sams et al., 2006; Velde et al., 2005; Wohlfarth et al., 2013) are the result and may contribute to the effectiveness of animal‐assisted treatments (Lundqvist et al., 2017).

### Therapy commitment and drop‐out rates

‘Drop‐out’ refers to prematurely quitting an intervention and constitutes a major problem for the effectiveness of all therapeutic treatments. In 1980, the National Institute of Mental Health (NIMH) estimated the drop‐out rate of psychotherapy between 30% and 60%. The first large meta‐analysis was conducted in 1993, compared 125 psychotherapy studies, and resulted in an average drop‐out rate of 46.8% with a 95% confidence interval (CI) of 42.9%–50.8% (Wierzbicki & Pekarik, 1993). Similarly, Kazdin and Mazurick (1994) reported in their meta‐analysis a drop‐out between 40% and 60%, while even higher drop‐out rates have been reported in child and adolescent psychotherapy (Deakin et al., 2012).

Over the last decades, the acceptance of psychotherapy in society has grown and the documentation of drop‐out rates in psychotherapy research has become standard. In 2012, a meta‐analytic comparison of 669 studies including 84,000 adult patients resulted in a mean drop‐out rate of 19.7% (95% CI: 18.7%, 20.7%) and provided insights into moderating factors such as manualization, clients diagnosis, age and sex (Swift & Greenberg, 2012). Three years later the across study drop‐out rate for 115 cognitive‐behavioural therapy studies was estimated to 26.2% (95% CI: 23.1%, 29.7%) by Fernandez et al. (2015). Finally, Linardon et al. reported in 2018 a mean drop‐out of 20.6% (95% CI: 17.4%, 24.2%) based on 72 individual therapy studies of different disorders. Overall, drop‐out rates decreased but rates around 20% leave still room for improvement.

### Aim of this meta‐analysis

Animal‐assisted psychotherapies have been proposed to reduce drop‐out rates (Jones et al., 2019), as they are highly accepted by patients (Hartfiel et al., 2017; Nurenberg et al., 2015; Schramm et al., 2015; Uhlmann et al., 2019). Support for this assumption comes from the observation that patients prefer animal‐assisted therapies over alternative therapies when offering multiple options (Dravsnik et al., 2018; Holcomb & Meacham, 1989; Trotter et al., 2008), patients are more willing to enter therapy when animals are involved (Ladner, 2016), and rising demand for animal‐assisted therapy has been demonstrated (Buck et al., 2017). Also, the general public opinion about animal‐assisted therapy is less stigmatizing compared to conventional therapy (Lass‐Hennemann et al., 2015) and first empirical comparisons suggested lower drop‐out in animal‐assisted psychotherapy than in conventional therapy (Bachi, 2012; Schramm et al., 2015; Uhlmann et al., 2019). However, systematic across study comparison of drop‐out rates in animal‐assisted psychotherapy is missing.

The present review and meta‐analysis close this gap by systematically summarizing empirical evidence on animal‐assisted psychotherapy and by comparing drop‐out rates meta‐analytically. On the basis of our results, we then highlight opportunities and challenges of animal‐assisted psychotherapy, propose subjects to future research and discuss differential indications.

## METHODS

### Definition of drop‐out

To allow for meta‐analytical comparison despite slightly varying definitions of drop‐out we considered drop‐out as what the authors of each study defined as such (Linardon et al., 2018). In cases without any explicit definition the following was used: ‘Drop‐out’ refers to the case ‘when a client unilaterally discontinues an intervention prematurely, before recovering from the problems that led him or her to seek treatment or before completing an intervention's specified protocol’, or both (Swift & Greenberg, 2014).

### Search strategy

All potential studies published until 23 August 2022 were retrieved and selected using the Preferred Reporting Items for Systematic Reviews and Meta‐Analysis (PRISMA, Page et al., 2021). The databases PubMed, PsycNET, ProQuest, EBSCOhost, Taylor & Francis Online, Web of Science and Google Scholar were searched through with the terms (animal OR dog OR canine OR horse OR equine OR pet OR Tier OR Hund OR Pferd OR hippo) AND (assisted OR facilitated OR tiergestützt) AND (dropout OR ‘drop out’ OR drop‐out OR attrition) AND (psychotherapy OR Psychotherapie). The term ‘failure’ was not included as predominantly finding reports of cardiac arrest. The identified references were screened for eligibility and both English‐ and German‐language studies were considered.

### Eligibility criteria

Studies were included in the meta‐analysis if (A) they were based on real animal‐assisted psychotherapy (instead of informal interventions including an animal, see below), and (B) drop‐out rates were reported. Beyond this, articles were excluded if they were (a) not available in English or German, (b) not peer‐reviewed, (c) no empirical study (e.g., reviews, books), (d) a meta‐analysis, (e) not focused on psychotherapy, (f) based on participants without psychological disorders, (g) based on sample sizes <4 (see Grawe et al., 2001; Wilkie et al., 2016), or (h) focused on animals for different reasons, for example, exposure‐treatment of animal phobia. If it was not possible to clearly identify all required information of a study, respective information was requested from the authors via email.

**FIGURE 1:**
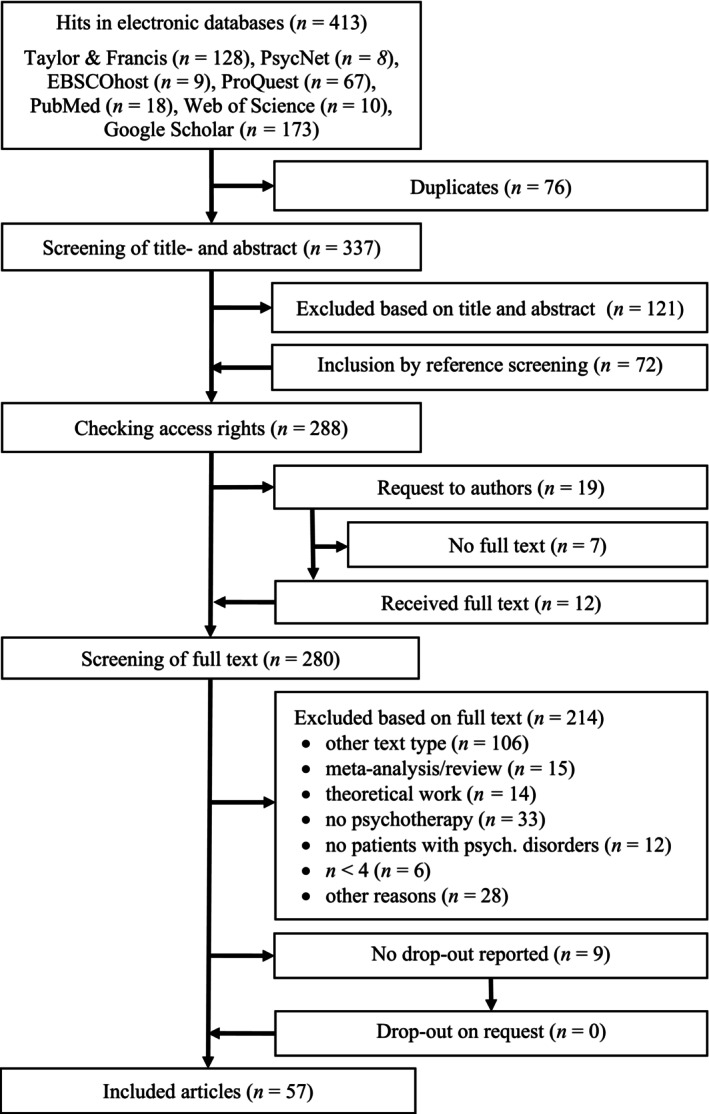
Flowchart of study selection process following the PRISMA guidelines.

To account for the important distinction between formal animal‐assisted psychotherapy and informal interventions referred to as animal‐assisted activities (Jones et al., 2019), we classified an intervention as psychotherapy if the authors used the term psychotherapy or if the paper indicates that the intervention was accompanied by a trained therapist and involved patients with psychological disorders. A flow chart showing the number of excluded studies in each step is illustrated in Figure 1.

### Data collection

For all included studies, the following basic variables were extracted: year of study publication, sample size *n* of the intervention group, absolute drop‐out, animal species used in the intervention, mental disorder to be treated, average duration of the intervention, participants sex, and age, as well as potential presence of any control group (see Appendix S1: *Basic Data*). The variable ‘animal species’ was categorized into ‘dog’, ‘horse’ and ‘x’ standing for animal species that cannot be assigned to dogs or horses, or multiple animal species were used simultaneously in an intervention. For the variable ‘mental disorder’ five classifications were defined [‘depression’, ‘schizophrenia’, ‘autism’, ‘trauma’, ‘addiction’] as well as the category ‘x’ for diagnoses or definitions that were too infrequent to form a separate group. Category x included attention deficit hyperactivity disorder (ADHD) (*m* = 1), forensic patients (*m* = 1), aggressive disorders (*m* = 2), vulnerable youth (*m* = 2) and heterogeneous diagnoses (*m* = 12).

### Data processing

As described in Borenstein et al. (2009), each original study's drop‐out rate was weighted on its sample size as well as on the total pool size (sum of all participants of all included studies). Specifically, the drop‐out rate per original study sample was determined as percentage (*M*_sample_) and then multiplied with the proportion between the size of this original study sample (*n*_sample_) multiplied with the number of original study samples included in this meta‐analysis (*m*) and the number of participants included in this meta‐analysis in total, that is, the total pool size (*n*_total_):

$$M_{\text{weighted}}=M_{\text{sample}}\times \frac{n_{\text{sample}}\times m}{n_{\text{total}}}.$$

To test the main hypothesis, that is, lower drop‐out in animal‐assisted psychotherapy than in conventional psychotherapy, a benchmark was required for drop‐out in conventional psychotherapy with which the drop‐out rate in animal‐assisted psychotherapy could be compared with. As the studies included in this meta‐analysis were much more heterogeneous than any single comparison sample for conventional psychotherapy would be and, thus, the requirement of variance homogeneity would not be fulfilled (see Results), we refrained from using a single meta‐analysis as comparison sample. Instead, we considered three representative meta‐analyses that collectively comprised *k* = 861 study samples showing similar diversity in treatments as the studies identified as eligible for our meta‐analysis. These were Swift and Greenberg (2012), Fernandez et al. (2015), and Linardon et al. (2018). With the meta‐analysis by Swift and Greenberg (2012), we include the most comprehensive meta‐analysis on drop‐out in psychotherapy for adults, while Fernandez et al. (2015) refer specifically to cognitive‐behavioural therapy, and Linardon et al. (2018) to interpersonal psychotherapy, while the latter two therapeutic approaches were most often used as basis for the development of animal‐assisted interventions. Our benchmark, the average drop‐out rate of conventional psychotherapy was calculated as weighted average drop‐out rate from the three respective meta‐analyses:

$$\mu =\frac{\left(M1\times k1\right)+\left(M2\times k2\right)+\left(M3\times k3\right)}{k1+k2+k3}$$

with *M* representing the meta‐analytically derived drop‐out rate and *k* the sample size of that meta‐analysis (number of included studies). The resulting population parameter *μ* was used in the following statistical analyses as benchmark with which we compared the meta‐analytically derived across sample drop‐out rate of animal‐assisted psychotherapy studies.

### Data analysis

For each included intervention group of a study, a separate data file was created. The software Jamovi (version 1.6, The jamovi project, 2022) was used for descriptive analyses and graphical illustrations, while inference statistics were performed with Microsoft Excel (version 5.13.7, Microsoft Corporation, 2023). Specifically, to test the main hypothesis (i.e., lower drop‐out in animal‐assisted psychotherapy than in conventional psychotherapy) a one‐sample *t*‐test with a 95% confidence interval was calculated as the assumption of variance homogeneity (homoscedasticity) as required for a two‐sample *t*‐test was not met (see Results section). *p*‐Values < .05 were considered as indicating statistical significance. Cohen's *d* (Cohen, 1988) was used as effect size measure, and post‐hoc power analyses were performed with G*Power (version 3.1, Faul et al., 2007). The requirements for statistical tests were evaluated in advance with appropriate procedures.

### Assessment of study design quality

To systematically evaluate the quality of included studies, we initially intended to use the Cochrane risk of bias tool 2.0 (Chandler et al., 2016) or the Study and Implementation Assessment Device (DIAD, Valentine & Cooper, 2008). However, the Cochrane risk of bias tool seemed inappropriate due to its strong focus on randomized controlled trials in medical research and the DIAD, although more suitable for psychological studies and allowing to adapt rating categories to the field of study (e.g., Linhardt et al., 2022; Pfeiffer et al., 2024), could not be used due to the insufficient reporting of study design characteristics and the heavily individualized research designs as common in animal‐assisted psychotherapy studies. Therefore, we developed an own rating system that enabled at least a systematic comparison of design quality among the here included studies and provided insights into common reporting practices. Firstly, we defined six categories: (1) report of information about intervention, (2) report of information about participants, (3) control conditions, (4) description and justification of operationalization, (5) report of statistical data and (6) Open Science practices. Secondly, we defined variables to operationalize each category and thirdly, each study was rated based on these variables. The overall rating of study quality was then calculated as sum of each study's score on each category.

The extracted variables contributing to our rating were (listed per category): (1) Type of animal used in the intervention, number/frequency of sessions, duration of one session, time period during which the whole intervention was completed, individual or group therapy, in‐ or outpatient setting, intervention following a standardized plan (Yes, No), (2) mental disorder of participants, participants age and gender, (3) implementation of any control condition (Yes, No), randomized group assignment (Yes, No), (4) description and justification of operationalization, (5) report of *p*‐values, test variables, standard deviations and effect sizes, (6) preregistration (Yes, No) and whether all data are freely available (Yes, No).

The following additional variables were not included in the quality rating but were extracted for more comprehensive insights about the research designs of included studies: blinded or unblinded data analysis, within‐ or between subject study design, whether any statistical test was performed, and whether additional online material was provided. For more detailed information about the quality rating procedure and variables of each category see Appendix S1: *Quality Rating Data*.

### Missing data

Since only studies with all variables relevant to the analysis were included, there were no missing values regarding drop‐out. Missing data collected in the additional variables is marked as such by leaving the corresponding fields blank in the tables.

## RESULTS

### Descriptive results

Sixty data sets from 57 studies with a total subject number of *n* = 1350 (mean data set sample size: *M* = 22.5, *SD* = 20.3) were identified as eligible for meta‐analytical comparisons. In studies with more than one intervention group, a separate data set was created for each group. The year of publication ranged between 1989 and 2022, with an increasing number of publications over the past 20 years and all full‐texts were accessible in English. The largest sample group comprised *n* = 121 patients, while the smallest group, as required by our inclusion criteria, included *n* = 4 patients (Table 1). Dogs were used as therapeutic animals in *m* = 18 samples, horses in *m* = 33. ‘Trauma’ was the most frequent mental disorder category followed by ‘depression’ and ‘schizophrenia’ (Table 2; for more detailed information about types of animals and mental disorders see Appendix S1: *Quality Rating Data*).

**TABLE 1:** Included studies.

Authors (year)	*n*	Drop‐out	Drop‐out [%]	Animal species	Mental disorder	Duration [h:min]	Gender [% female]	Age	Control group	Quality rating [0;10]
Antonioli and Reveley (2005)	15	2	13.33	x	Depression	10:00	93.00	41.0	Yes	4
Atherton et al. (2020)	10	0	.00	Horse	Addiction	9:00	40.00	10.5	No	4
Bachi et al. (2012)	14	0	.00	Horse	x	24:10		16.0	Yes	3
Balluerka et al. (2015)	43	2	4.65	x	x	23:00	48.72	15.7	Yes	6
Barak et al. (2001)	10	0	.00	Horse	Schizophrenia	208:00	70.00	79.1	Yes	5
Bass et al. (2009)	19	0	.00	x	Autism	12:00	10.53	6.9	Yes	7
Berget et al. (2008)	60	19	31.67	Horse	x	72:00	65.56	34.7	Yes	7
Burton et al. (2019)	10	0	.00	x	Trauma	6:00	20.00	48.0	Yes	5
Byström et al. (2019)	9	0	.00	Horse	Autism	100:00	22.22	7.0	No	4
Calvo et al. (2016)	16	2	12.50	Horse	Schizophrenia	40:00	25.00	47.8	Yes	6
Cerino et al. (2011)	24	0	.00	Dog	Schizophrenia	40:00			No	2
Collingwood et al. (2021)	7	0	.00	Horse	x	150:00	0.00		No	2
Contalbrigo et al. (2017)	12	3	25.00	Horse	Addiction	20:00	0.00	35.5	Yes	5
Dietz et al. (2012)	121	0	.00	Dog	Trauma	3:00	93.39	11.3	Yes	6
Earles et al. (2015)	16	0	.00	Horse	Trauma	8:00	75.00	51.3	No	3
Fisher et al. (2021)	63	5	7.94	Horse	Trauma	8:00	37.00	50.0	No	5
Folse et al. (1994)	11	2	18.18	Dog	Depression	5:15	77.27	21.0	Yes	3
	13	1	7.69	Dog	Depression	5:15	77.27	21.0	Yes	3
Gabriels et al. (2012)	42	4	9.52	Horse	Autism	10:00	16.67	8.7	Yes	5
Gatti et al. (2020)	18	4	22.22	Horse	Addiction	30:00	55.56		Yes	7
Johnson et al. (2018)	28	9	32.14	Horse	Trauma		15.79	54.4	Yes	5
Keino et al. (2009)	4	0	.00	Horse	x		.00	7.0	No	2
Kemp et al. (2014)	15	0	.00	Horse	Trauma	14:15	60.00	9.8	No	4
	15	0	.00	Horse	Trauma	14:15	100.00	15.5	No	4
Kendall and Maujean (2015)	16	4	15.00	Horse	x	10:00	31.25	15.4	Yes	5
Kern et al. (2011)	24	4	16.67	Horse	Autism	12:00	25.00	7.8	Yes	3
Kern‐Godal et al. (2016)	8	0	.00	Horse	Addiction	12:00	50.00	24.8	No	1
Kloep et al. (2017)	13	0	.00	Dog	Trauma		30.77	40.7	No	4
Klontz et al. (2007)	49	18	36.74	Horse	x	28:00	70.97	44.7	No	4
Kovács et al. (2004)	7	0	.00	Dog	Schizophrenia	32:20	57.14	43.6	No	1
Lanning and Krenek (2013)	13	6	46.15	Horse	Trauma	36:00	23.08	35.5	No	1
Marr et al. (2000)	18	0	.00	x	Addiction	20:00	30.00	41.5	Yes	6
McCullough et al. (2015)	12	1	8.33	Horse	Trauma	13:00	45.45	13.7	No	3
Memishevikj and Hodzhikj (2010)	4	0	.00	Horse	Autism	5:00	50.00	9.3	No	3
Muela et al. (2017)	60	8	13.33	x	x	34:00	48.08	15.00	Yes	5
Muela et al. (2019)	19	0	.00	Dog	Trauma	13:00	32.58	8.9	No	5
Mueller and McCullough (2017)	36	11	30.56	Horse	Trauma	20:00	16.67	15.0	Yes	5
Nathans‐Barel et al. (2005)	10	0	.00	Dog	Schizophrenia	10:00	40.00	39.9	Yes	5
Nurenberg et al. (2015)	24	1	4.17	Horse	x	8:20	25.00	44.3	Yes	6
	25	2	8.00	Dog	x	8:20	44.00	45.0	Yes	6
Prothmann et al. (2006)	61	20	32.79	Dog	x	2:30		14.9	Yes	5
Redefer and Goodman (1989)	12	0	.00	Dog	Autism	6:00	25.00	7.5	No	2
Roberts and Honzel (2020)	37	3	8.11	Horse	x	7:00	24.32	15.1	No	3
Romaniuk et al. (2018)	25	0	.00	Horse	Trauma		24.00	50.3	Yes	5
Saggers and Strachan (2016)	11	0	.00	Horse	x	16:00	54.55	11.4	No	2
Sams et al. (2006)	22	0	.00	x	Autism	5:00		9.6	No	4
Schramm et al. (2015)	6	0	.00	x	Depression	20:00		47.7	No	8
Schramm et al. (2022)	31	2	6.45	x	Depression	22:30	80.60	47.5	Yes	3
Schroeder et al. (2018)	9	1	11.11	Horse	Trauma	27:00	100.00	44.0	No	3
Schuck et al. (2015)	12	0	.00	Dog	x	54:00	17.00	8.0	Yes	7
Schultz et al. (2007)	63	14	22.22	Horse	x	19:00	41.00	10.9	No	4
Stefanini et al. (2015)	17	0	.00	Dog	x	9:00	52.94	15.4	Yes	6
Stefanini et al. (2016)	20	0	.00	Dog	Schizophrenia	7:30	55.00	15.2	Yes	4
Tóthné (2018)	7	0		Dog	x	22:30	28.57	10.0	No	3
Trujillo et al. (2020)	14	0	.00	Dog	x	10:00	21.00	13.7	Yes	6
Villalta‐Gil et al. (2009)	12	0	.00	Dog	Schizophrenia	18:45	9.30	49.1	Yes	8
Wharton et al. (2019)	27	2	7.47	Horse	Trauma	8:00	22.00	51.0	No	4
Whittlesey‐Jerome (2014)	7	1	14.29	Horse	Trauma	16:00	100.00	47.0	Yes	4
Willmund et al. (2021)	20	0	.00	Horse	Trauma		5.00	41.20	Yes	5
Yorke et al. (2013)	4	0	.00	Horse	Trauma	6:00		9.0	No	3

*Note*: Sample size refer to the sizes of the respective intervention groups. ‘x’ in the columns animal species and mental disorder indicates cases in which different animal species/mental disorders were used/treated or cases in which the expressions of these variables were too low in number to create a separate group. ‘Duration’ refers to the mean duration of the intervention across all treated patients. In the other columns, an empty cell depicts that no data on these variables was reported in the respective study. All data were rounded to the indicated number of decimals. The last column lists each study's sum score resulting from the study design quality rating (see Methods). For more detailed information about types of animals, mental disorders and further characteristics of study designs see Appendix S1: *Quality Rating Data*.

**TABLE 2:** Varying drop‐out rates for different mental disorder groups.

Mental disorder		Drop‐out [%] (weighted)		
	*m*	*M*	*SD*	*SE*
Trauma	18	8.9	15.2	3.6
Autism	7	5.1	8.7	3.3
Schizophrenia	7	1.3	3.4	1.3
Depression	5	6.2	4.0	1.8
Addiction	5	6.2	8.7	3.9
Other	19	21.3	32.2	7.4

*Note*: ‘*m*’ refers to the number of samples on the respective disorder. ‘Other’ includes groups with mixed diagnoses (*m* = 12) or mental disorders that were too few in number to be considered as separate group, such as ADHD (*m* = 1), forensic patients (*m* = 1), aggressive disorders (*m* = 2) and vulnerable youth (*m* = 2). For more detailed information about mental disorders see Appendix S1: *Quality Rating Data*.

The average duration of animal‐assisted interventions was 24 h and 38 min with a high standard deviation of 36 h 51 min. The shortest intervention lasted only 2.5 h (Prothmann et al., 2006), while the longest comprised 208 h, with a weekly 4‐h treatment over 1 year (Barak et al., 2001). For 54 of 57 data sets, sex ratios were reported. Overall, 42.3% of the patients were female. The mean age of patients was 27.4 years (*SD* = 18.0), but three of the included studies provided no age information (Cerino et al., 2011; Collingwood et al., 2021; Gatti et al., 2020). Specifically, the youngest study sample had an average age of 7.0 years, while the oldest sample was on average 79.1 years old. Adult patients were investigated in 28 data sets, while children and adolescents were included in 29 data sets. Finally, of the 60 original study data sets 33 resulted from a study design including a control group.

**FIGURE 2:**
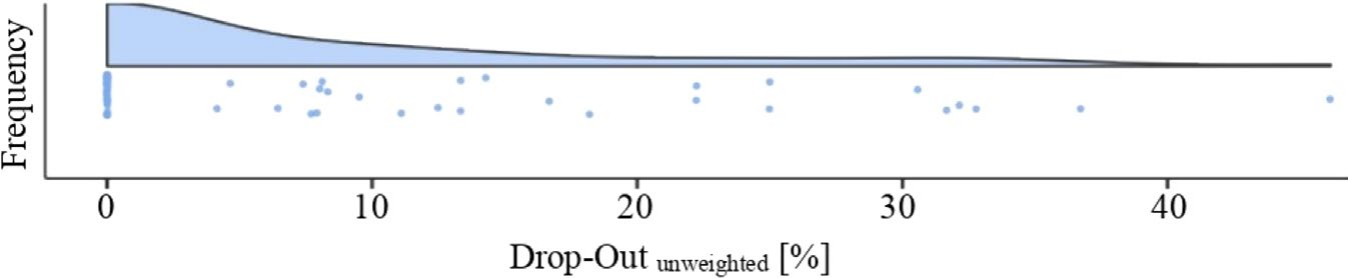
Frequency distribution of original study drop‐out rates. Each dot represents the true drop‐out from a single sample of all included animal‐assisted psychotherapy studies.

The drop‐out rate across all data sets weighted by the sample sizes was 11.2% (*SD* = 21.2). Note that the standard deviation was scaled by the weighting (${\mathrm{SD}}_{\text{non}-\text{weighted}}=11.6$) and that Figure 2 depicts the frequency distribution of all considered drop‐out rates. Taking potential moderator variables into consideration, the weighted drop‐out rate was 7.4% (*SD* = 19.7) for dog‐assisted psychotherapy and 11.9% (*SD* = 20.8) for equine‐assisted psychotherapy. The five categories of mental disorders differed also descriptively from each other in the mean drop‐out values (see Table 2).

### Study design quality rating

Supporting the validity of our rating procedure, the resulting study design quality scores varied markedly between different studies (range: 1–8, *M* = 4.32, *SD* = 1.68) and were approximately normally distributed (see Table 1 and Appendix S1: *Quality Rating Statistics*). The category that was rated best was (4) ‘description and justification of operationalization’ with only nine of the 60 included studies that lacked sufficient information (range: 0–1; *M* = .85; *SD* = .36). The categories (5) ‘report of statistical data’ (range: 0–2; *M* = .98; *SD* = .79) and (1) ‘report of information about the intervention’ (range: 0–2; *M* = .93; *SD* = .73) followed and revealed problems especially with respect to the reporting of effect size measures and the lack of standardized intervention plans. Control conditions were implemented in 33 studies with 16 of them performed randomized group assignment (category 3: range: 0–2; *M* = .82; *SD* = .83). Category (2) ‘report of information about participants’ revealed serious shortcomings (range: 0–1; *M* = .67; *SD* = .48), especially with respect to missing group‐specific (control vs. intervention group) information on basic demographical variables. ‘Open Science practices’ was the category with the lowest ratings (range: 0–2; *M* = .07; *SD* = .25). Specifically, only four out of the 60 included studies were preregistered (Burton et al., 2019; Fisher et al., 2021; Gatti et al., 2020; Schramm et al., 2022) and not a single study made their data freely available. All details concerning the rating procedure as well as all category‐specific scores are listed in the Appendix S1: *Quality Rating Data*.

### Inference statistical comparison of drop‐out rates

#### Statistical requirements

Statistical assumptions for the initially planned two‐sample *t*‐test (normality and homoscedasticity) were evaluated comparing the here‐derived across study mean drop‐out rate with the mean drop‐out rate of conventional therapy. Drop‐out has a naturally right‐skewed distribution and is therefore not normally distributed. However, since a total of *m/k* > 30 observations were available, a normal distribution can be assumed based on the central limit theorem, irrespective of the parent sample (Bortz & Schuster, 2010). As the variance in the present study is higher than the variance in the three meta‐analyses on conventional therapy (*Var* = 449.4 in our meta‐analysis vs. *Var* [173.5, 359.0] in the three conventional therapy meta‐analyses), the requirement of variance homogeneity (homoscedasticity) for two‐sample *t*‐tests was not met. However, a violation is only problematic if the larger sample is characterized by lower variance (Bortz & Schuster, 2010), which is not the case in our investigation. Therefore, we considered a one‐sample *t*‐test as appropriate alternative with the population parameter *μ* that was derived from the three meta‐analyses on conventional psychotherapy.

### Benchmarking the drop‐out rate in conventional psychotherapy

**FIGURE 3:**
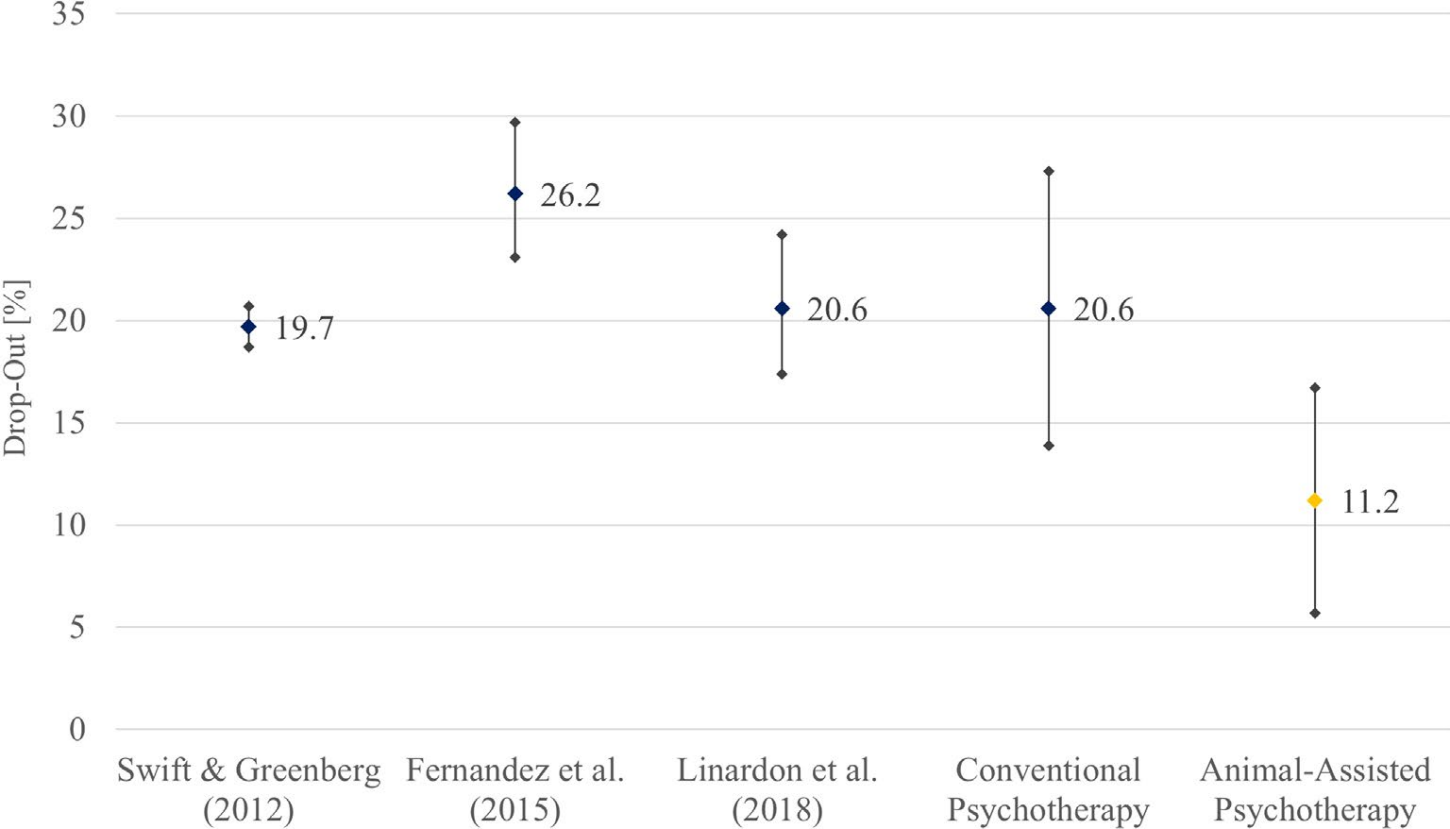
Cross‐study drop‐out rates of conventional versus animal‐assisted psychotherapy. Across study mean drop‐out rates with 95% confidence intervals (CI) from three large meta‐analyses comparing drop‐out rates of conventional psychotherapy (graphs 1–3, blue) and their weighted summary (graph 4, blue) compared to the meta‐analytically derived across study drop‐out rate of animal‐assisted psychotherapy (graph 5, yellow).

To statistically assess our main hypothesis, that is, lower drop‐out rates in animal‐assisted psychotherapy than in conventional psychotherapy, an estimate of the drop‐out rate in conventional psychotherapy was created. Specifically, we averaged across the means of Swift and Greenberg (2012): *M* = 19.7%, *k* = 669, Fernandez et al. (2015): *M* = 26.2%, *k* = 115, and Linardon et al. (2018): *M* = 20.6%, *k* = 77. The resultant across meta‐analyses mean was *μ* = 20.6%, 95% CI [13.9%, 27.3%] (see Figure 3). This value serves as benchmark with which the across study average drop‐out rate of animal‐assisted psychotherapy was compared with.

### Lower drop‐out in animal‐assisted psychotherapy

Across all included data sets, the weighted mean drop‐out rate for animal‐assisted psychotherapy was 11.2% (95% CI [5.7%, 16.7%]). This was significantly lower than the across meta‐analyses mean of conventional therapy (*μ* = 20.6%) at a significance level of *α* = .05 with *t*(59) = −3.46, *p* = .0005, *d* = −.446, corresponding to a power of 1 – *β* = 96.2%, *t*(59) = 1.67 to detect an effect of this size.

### Moderating influences on drop‐out rates in animal‐assisted therapy

The extremely large heterogeneity as well as the rather small and unequally sized samples of the original studies belonging to one moderator variable category (e.g., animal species or type of mental disorder; see Methods) prevented us from conducting the initially planned statistical moderator analyses. Therefore, only descriptive differences are reported (Tables 1 and 2) and cautiously discussed with respect to potential moderators.

## DISCUSSION

This quantitative meta‐analysis aimed at shedding light on the question whether the inclusion of animals in psychotherapy could reduce drop‐out rates. Therefore, the drop‐out rates of 60 animal‐assisted psychotherapy samples from 57 empirical studies were compared systematically. Further, we aimed at gaining insights into potential moderators of drop‐out in animal‐assisted psychotherapy with the aim of identifying mechanisms relevant for exiting a therapy and offering starting points for future research in this evolving field. In sum, our results suggest that animal‐assisted psychotherapy could serve as a means to increase commitment and participation, providing insights into potential mechanisms leading to lower drop‐out in psychotherapy. However, we also revealed serious problems in study design quality that hinder drawing strong conclusions and need to be overcome by future research.

### Lower drop‐out in animal‐assisted psychotherapy

The meta‐analytically derived across study drop‐out rate in animal‐assisted psychotherapy was significantly smaller than that derived from three large‐scale meta‐analyses focusing on conventional psychotherapy. The 95% confidence interval of the weighted drop‐out in animal‐assisted psychotherapy does not overlap with any of the 95% confidence intervals of the three conventional therapy meta‐analyses, but with that of the weighted average *μ* of the three meta‐analyses, due to high heterogeneity. Thus, although future meta‐analyses that consider a larger number of studies are essentially required, our meta‐analysis provides a first hint suggesting that the use of animals in psychotherapy could lead to fewer discontinuations.

Various aspects of animal‐assisted psychotherapy might have contributed to the relatively low drop‐out rates. Firstly, there are multiple positive characteristics that people often associate with animals such as their ability for positive and valuable non‐verbal communication (Karol, 2007; Schüppel, 2012; Velde et al., 2005) and the assumption that animals act free of any judgement (Klontz et al., 2007; Ladner, 2016; Schramm et al., 2015; Vidrine et al., 2002; Wilson et al., 2017). Secondly, the mere presence of animals seems to induce positive emotions in humans (Burton et al., 2019). This might, among other things, also lead clients to enjoy going to therapy and to build positive associations with respect to both the therapy and the therapist. Thirdly, the mere presence of animals has been demonstrated to reduce stress by, for example, the release of Oxytocin (Beetz et al., 2012; Handlin et al., 2011; Odendaal, 2000), which in turn could have an effect on improved absorption readiness during therapy. Lastly, various therapeutic interventions can be clarified or illustrated through interactions with animals such as clearly expressing own thoughts and feelings. Animals do only react intendedly when they have the chance to detect the patient's need (expressions must be strong enough), but animals do not laugh or judge when expressions are too direct or too powerful (Lee et al., 2016). In this regard, animals provide also the possibility to make new corrective experiences in certain areas important for the patient, for example, to express own needs without being laughed at or to enjoy physical touch without breaking ethical or sexual boundaries (Wesley et al., 2009).

### Moderators

Although study heterogeneity and the lack of a sufficient number of studies did not allow us to statistically analyse potential moderating influences on drop‐out rates, notable descriptive differences were observed (a) between the use of dogs and horses and (b) between different disorder groups. The weighted mean drop‐out rate in dog‐assisted psychotherapy (7.4%) was lower than that of equine‐assisted psychotherapy (11.9%). Most likely, this may be caused by the fact that participating in therapy accompanied by dogs generally involves less effort for patients than visiting a farm outside of a city as required for equine‐assisted psychotherapy (Coakley & Mahoney, 2009; Tyler, 1994). In general, multiple factors might influence which type of animal suits best for a specific therapeutic purpose. Personal preferences of the patient, the type of disorder and setting are only some of these factors that might be valuable to consider. However, as long as no further meta‐analytical evidence is available, individualized decisions may be preferable concerning the question of which type of animal might be best suited for a particular purpose.

Similarly, as for the moderator variable ‘animal species’, also the clustered groups of ‘patient's disorder’ were too small to allow for reliable inferential statistics. Nevertheless, we observed that studies focusing on a specific disorder group had lower drop‐out rates than those with mixed or undefined groups (Table 2). A potential reason for this observation may be that disorder‐specific mechanisms can be better addressed in homogeneous groups, which in turn reduces symptoms more effectively and leads to higher commitment to the therapy.

Considering both moderators together, it can be suspected that some animal species are better suited for certain patient groups than for others. Horses, for example, are frequently used in the treatment of PTSD due to their characteristics as flight and non‐harassing animals (Kemp et al., 2014; Klontz et al., 2007; McCullough et al., 2015; Schultz et al., 2007), while dogs due to their high prompting character are well suited to teach people to set their own boundaries, which is for example often affected in autism, and can provide self‐efficacy experiences by showing boundaries to the dog (Seitz et al., 2017).

To conclude, the questions of whether all or only particular psychological disorders might benefit from including an animal in the therapeutic setting as well as which animal species might be best suited for a given disorder are important aspects for future investigations. As far as a markedly increased number of original studies is available, future meta‐analyses should be conducted to provide more comprehensive insights into the role of potential moderators such as animals used, disorders treated, intervention duration and patient demographics. The present work provides a baseline and offers hypotheses that might be worth to address in future research, such as, that the inclusion of horses in frequently applied trauma therapy might increase its effectiveness and significantly reduce drop‐out.

### Strengths and limitations

The strength of the current study is the exhaustive literature search that allowed for statistical comparisons of drop‐out rates in animal‐assisted psychotherapy and the fact that it is the first endeavour addressing this topic systematically. However, the quality of a meta‐analysis is always restricted by the quality of the original studies. The studies considered here were not only very heterogeneous, but often also characterized by serious problems regarding proper research design. Some of these problems could even bias study results (and drop‐out rates) and may present a threat for good scientific practice (e.g., lack of standardized plans, missing Open Science practices). This has been criticized frequently (Kamioka et al., 2014; Lee et al., 2016; O'Haire, 2013; Schramm et al., 2015; Serpell et al., 2017) and prevented us from drawing strong conclusions as well as from conducting the planned moderator analyses. The sample sizes in animal‐assisted psychotherapy studies are often small and patients with heterogeneous diseases and of different settings (in‐ and outpatient) are mixed. Inpatient treatments are associated with lower drop‐out rates and could, thus, have induced a bias as we did not differentiate between in‐ and out‐patient treatments. Moreover, information about sample characteristics and intervention sequences was not always precise or even lacking. This is highly problematic as, for example, the total length of an intervention as well as, in case of multi‐session interventions, the temporal intervals between different sessions might impact drop‐out rates and, thus, an intervention's effectiveness.

Additional limitations refer to the comparison of the here derived across study drop‐out rate for animal‐assisted psychotherapy with the drop‐out rates for conventional psychotherapy. As we could not access the original study samples included in the three large meta‐analyses on conventional psychotherapy (see study quality rating: lack of Open Science practices), we could not precisely identify whether and if so to which extent the included samples of the three averaged meta‐analyses on conventional psychotherapy overlapped and we were only able to perform the comparison indirectly. This could potentially have affected the weighting procedure in the averaging process. Although it has to be acknowledged, that Open Science practices were rather uncommon during the time the first animal‐assisted psychotherapy studies have been published, another danger that comes with lacking Open Science practices is that without preregistration, we cannot exclude the possibility that the reported effects do not actually represent an optimistic overestimation – the reported drop‐out rates an optimistic underestimation. Most critical, some animal‐assisted psychotherapy studies included in our meta‐analysis report the absence of any drop‐out, which might seem rather unrealistic. However, there might exist methods for enhancing patient's commitment, including a very familiar atmosphere in small‐sample studies, such that we cannot differentiate between the possibility that the absence of any drop‐out is actually true or represents ‘only’ a lack of reporting standards. In the future, this problem has to be overcome by more strictly following existing reporting standards and by the implementation of Open Science practices such as the preregistration of hypotheses and definitions of drop‐out or the use of registered reports that facilitate the publication of null‐ or contradictory findings.

Finally, the large difference in the variance of drop‐out rates in the studies included in our meta‐analysis and the variance of drop‐out rates in the meta‐analyses on conventional therapy may have affected the validity of the test statistic. These issues need to be overcome in future meta‐analytical efforts on the basis of a larger empirical background consisting of studies with higher research design quality and by direct comparison of original study samples for animal‐assisted psychotherapy with original study samples from conventional psychotherapy within a single analysis.

### Perspectives for future research

The work presented here proposes on the one hand that the inclusion of an animal in the therapeutic setting could reduce drop‐out rates, on the other hand, it also highlights methodological shortcomings of existing studies, challenges and open research questions that should be addressed in future studies. The main prerequisite for the use of animals in psychotherapy is, of course, the willingness of the individual patient to involve a particular animal. Personal preferences and past experience play a major role, and studies must be conducted clarifying differential indication. This also raises the question, of whether animal‐assisted psychotherapy is only effective for people who like animals. Although, this seems intuitively plausible, no such influences have been reported in Schneider and Harley (2006), highlighting the need for further investigation. Also, it must be carefully analysed to what extent the effects go beyond self‐fulfilling prophecies due to positive patient attitudes, or whether the inclusion of animals can also have negative effects on the therapy outcomes for example due to distraction. Moreover, standardized manuals for animal‐assisted psychotherapy and concepts of how to implement animal‐assisted therapy in established therapy care plans are today only barely available and should, thus, be developed in the future. This requires an empirical and theoretical foundation.

Further, it is necessary to investigate outcome predictors and to define indications and contraindications. Beyond the contradiction of animal phobia (Morrison, 2007; Wesley et al., 2009), the main currently known aspects are risks for the safety of the patients and the potential infection by zoonotic diseases (Coakley & Mahoney, 2009; Morrison, 2007), the latter of which can be effectively prevented by vaccinating the animals. In contrast, also patients who could endanger the welfare of the animal (e.g., due to frustration and limited impulse control), should also be excluded from this therapy method if the safety of the animal cannot be guaranteed (Wesley et al., 2009). Animal welfare must also be taken into account in all other areas since a well‐balanced animal is the basis of every animal‐assisted psychotherapy to be effective. This includes, for example, appropriate breaks and low patient fluctuation as well as outside therapy species‐typical activities (Fine, 2015). These aspects should therefore be taken also into account in all future research endeavours.

Further concerns are the training of the animals, cost issues, and the specific setting. While, for example, Coakley and Mahoney (2009) describe dog‐assisted psychotherapy as a ‘low tech ‐ low cost’ intervention, horses are expensive and time‐consuming (Tyler, 1994). Concerning the training of therapeutic animals there is some evidence suggesting that the individual character of the animal plays a significant role (Bachi, 2012) and that excessive training is inadvisable, as dressage could suppress the authentic reaction of animals (Topel, 2014). However, to the best of our knowledge until today no study has attempted to systematically evaluate the training in the field of animal‐assisted psychotherapy. Also, the necessary qualification for therapists remain unclear, and whether individual or group therapy proves to be more effective or on which factors this depends. Finally, there is widespread consensus to not purely rely on animal‐assisted psychotherapy, but to use it as a complement to established therapy methods which is most extensively realized in inpatient settings (Dietz et al., 2012; Nimer & Lundahl, 2007; Reichert, 1998; Seitz et al., 2017; Selby & Smith‐Osborne, 2013; Wohlfarth et al., 2013). However, the effects of these simultaneously attended therapies may interact with the effects and commitment to the animal‐assisted therapy, ultimately influencing effectiveness and drop‐out rates. Therefore, a detailed coding and extraction of additional variables concerning the setting and further therapies is another adaption to be realized in future meta‐analyses.

Overall, our report highlights the pressing need for more systematic investigations in the field of animal‐assisted psychotherapy – a topic even more important considering its potential to reduce drop‐out.

## CONCLUSION

The drop‐out of patients in psychotherapy constitutes a pressing issue for its effectiveness. This meta‐analysis is the first systematically assessing drop‐out rates in animal‐assisted psychotherapy. To this aim, 60 original study samples were compared and the resulting across study drop‐out rate was set in relation to the across‐study drop‐out rates reported in the three largest meta‐analyses on conventional psychotherapy. The resulting across study drop‐out rate was 11.2% suggesting a positive effect of the inclusion of animals on therapy commitment. However, insights into potential causes and underlying mechanisms remain mostly unclear as the number of available original studies is small, those were often characterized by serious problems with respect to proper research design quality, and overall characterized by enormous heterogeneity. Due to the intensive additional investment necessary for the well‐being of the animal and requirements for the therapeutic set‐up, animal‐assisted therapy will likely remain an add‐on that will rarely be used but could be beneficial in clearly defined cases. However, in those cases therapeutic animals may render therapy effective that may otherwise fail.

## AUTHOR CONTRIBUTIONS

**Linnea Seeger:** Conceptualization; methodology; data curation; formal analysis; writing – review and editing; visualization. **Andrea Kübler:** Conceptualization; writing – review and editing; supervision. **Kirsten Hilger:** Conceptualization; methodology; writing – original draft; project administration; supervision.

## FUNDING INFORMATION

The publication of this article was supported by the Open Access Fund of the University of Würzburg.

## CONFLICT OF INTEREST STATEMENT

The authors declare no conflicts of interest.

## ETHICS APPROVAL STATEMENT

Not applicable as no original data was acquired. However, it was ensured that all studies included in this meta‐analysis had ethics approval and ensured for informed consent.

## Supporting information


Appendix S1.

## Data Availability

All extracted data allowing for reproducibility of study findings were made available in the Open Science Framework (OSF) and can be accessed under: https://osf.io/729c8/.
